# Real-world evaluation of an evidence-based telemental health program for PTSD symptoms

**DOI:** 10.1038/s41598-024-83144-6

**Published:** 2025-01-03

**Authors:** Jocelynn T. Owusu, Lu Wang, Shih-Yin Chen, Robert E. Wickham, Scott T. Michael, Nazneen F. Bahrassa, Alethea Varra, Jennifer L. Lee, Connie Chen, Anita Lungu

**Affiliations:** 1 Lyra Health, 270 East Ln, Burlingame, CA 94010 USA; 2 Department of Psychological Sciences, Northern Arizona University, Flagstaff, AZ USA; 3 Private Practice, Burlingame, CA USA; 4 Department of Pediatrics, Emory University School of Medicine, Atlanta, GA USA

**Keywords:** Posttraumatic stress disorder, Depression, Mental health, Psychotherapy, Telehealth, Digital health, Psychology, Health care, Outcomes research

## Abstract

Blended care therapy (BCT), which augments live, video-based psychotherapy sessions with asynchronous digital tools, has the potential to increase access to evidence-based treatments for posttraumatic stress disorder (PTSD). However, its effectiveness in diverse, real-world settings is not well-understood. This evaluation aimed to assess clinical outcomes of a BCT program for PTSD symptoms. A retrospective cohort analysis was conducted of 199 adults who received an employer-offered BCT program for PTSD symptoms that delivered either cognitive processing therapy or prolonged exposure. PTSD symptom severity was regularly assessed using the PTSD Checklist for DSM-5 (PCL-5). Growth curve models were used to evaluate the trajectory of PTSD symptoms over the course of care, and an interaction term was added to assess outcomes by baseline PTSD symptom severity (i.e., PCL-5 ≥ 31 versus PCL-5 < 31). End-of-care reliable improvement and recovery were evaluated. On average, participants with baseline PCL-5 < 31 exhibited statistically significant declines in PTSD symptoms during care, while participants with baseline PCL-5 ≥ 31 showed statistically significantly steeper initial declines in PTSD symptoms that became less pronounced over time. Overall, 82.91% of participants demonstrated either reliable improvement or recovery in PTSD symptoms. This evaluation suggests BCT for PTSD symptoms can be beneficial in real-world settings. Future research should perform large-scale evaluations.

**Supplementary Information:**

The online version contains supplementary material available at 10.1038/s41598-024-83144-6.

## Introduction

Worldwide, approximately 70% of adults experience a traumatic event across their lifetime and the estimated lifetime prevalence of posttraumatic stress disorder (PTSD) in trauma-exposed adults is 5.6%^1^. In the U.S alone, one study estimated that over 14 million civilian adults experience PTSD across their lifetime^2^. PTSD is associated with debilitating outcomes, including higher occurrences of disability and suicidal behaviors^2–5^. PTSD also results in substantial economic costs, with excess costs of $34.8 billion that are attributable to PTSD-related presenteeism and absenteeism in the U.S^6^. Despite its significant burden, the estimated past-year PTSD treatment prevalence was only 24% among U.S. civilians with lifetime PTSD^7^. When evaluating lifetime treatment prevalence, it is estimated PTSD goes untreated in 40.6% of U.S. civilian adults and treatment seeking is delayed by an average of 4.5 years among those who do eventually receive care^2^.

Research supports the effectiveness of evidence-based care, such as Cognitive Processing Therapy (CPT) or Prolonged Exposure (PE), for the treatment of PTSD symptoms^8–11^, as well as for symptoms of depression in adults with PTSD^12,13^. Treatment efficacy has been observed in those with clinically elevated levels of PTSD, as well as those with subclinical symptoms who are experiencing distress^14^. However, there exist significant barriers to receiving evidence-based care for PTSD^15^. For one, there is an insufficient number of providers who are able to provide specialized care such as CPT and PE^15^, and PTSD treatment is less readily available in community and private settings, particularly relative to the U.S. Department of Veterans Affairs (VA) Healthcare system^15^. Time constraints, affordability, transportation issues, and the stigma of seeking care for PTSD symptoms are also barriers to care^16–19^. PTSD symptoms, particularly avoidant behaviors (e.g., avoiding symptom triggers), are also factors that prevent treatment-seeking^16,19^. Given the significant and impairing consequences of PTSD, there is a strong need for treatment models that can better meet the care needs of impacted populations.

Evidence-based PTSD treatment delivered via telehealth show promise in mitigating common treatment barriers (e.g., travel-related issues)^15,20^, show comparable effectiveness to in-person care for PTSD^15,21^, and report similar levels of treatment satisfaction^21,22^. While internet-based interventions for PTSD symptoms (e.g., internet-based cognitive behavioral therapy [iCBT]) have also been found to be effective and could increase the accessibility of treatment^23^, as a standalone care option, dropout rates for internet-based interventions can be high^24^. Additionally, treatment effect sizes are larger in iCBT interventions for PTSD that include therapist involvement compared to those without therapist involvement^23^. Internet-based interventions that are delivered in between therapy sessions could increase the benefits of therapy; for example, they could facilitate the application of skills in day-to-day life^25^. Unsurprisingly, internet-based tools have been proposed as an approach to deliver between-session support for those receiving telehealth-based care for PTSD^26^. Altogether, this evidence provides support for a care model that blends therapy with digital tools in order to maximize treatment benefits.

In light of possible benefits as an effective and efficient care delivery model, blended care therapy that combines face-to-face therapy with digital tools has been evaluated for several mental disorders^25^. A growing body of literature has associated blended care therapy with positive clinical outcomes for common mental disorders^25,27–29^. There is also evidence that a blended care model can be effective and efficient for PTSD symptoms^30,31^. For instance, a recent study among veterans who were exposed to trauma found evidence-based blended care therapy for PTSD (i.e. weekly therapy sessions and digital modules) was associated with significantly lower PTSD and depression symptoms at the end of care and at 3-month follow-up^30^, providing preliminary support for this treatment approach.

Although research has reported positive outcomes for blended care therapy, the evidence is limited for blended care programs treating PTSD symptoms, particularly in populations outside of the VA. Therefore, the primary objective of this real-world evaluation was to evaluate changes in PTSD symptoms experienced by clients participating in an evidence-based blended care trauma treatment program delivered via telehealth. Among clients also presenting with clinically elevated depression symptoms, a secondary objective was to assess end-of-care changes in depression symptoms. This study preliminarily hypothesized participants receiving the evidence-based blended care trauma treatment program would experience significant improvements in clinical symptoms over the course of care and at the end of care.

## Results

Table 1 describes participants’ baseline characteristics. On average, participants completed 7.97 (SD: 2.95) therapy sessions, and the average duration of care in the BCT Trauma program was 9.98 (SD: 4.46) weeks. Overall, 77.39% of participants attended all scheduled therapy sessions, 19.60% missed one session, 2.51% missed two sessions, and 0.50% missed three sessions. Upon stratification by treatment type, 78.18% of participants who received CPT completed all scheduled therapy sessions, and 76.40% of participants who received PE completed all scheduled therapy sessions.

**Table 1:** Participant characteristics (N = 199).

Participant characteristics	Distribution
Age, mean (SD)	34.07 (9.26)
Gender, n (%)	
Female	170 (85.43)
Male	23 (11.56)
Other/missing	6 (3.02)
Race and ethnicity, n (%)	
White	108 (54.27)
Hispanic or Latino	24 (12.06)
Asian or Pacific Islander	15 (7.54)
Black or African American	10 (5.03)
Multiple	27 (13.57)
Other	10 (5.03)
Prefer not to disclose/unknown	5 (2.51)
Employee status, n (%)	
Employee	155 (77.89)
Dependent	32 (16.08)
Missing	12 (6.03)
Baseline PCL-5 ≥ 31, n (%)	112 (56.28)
Baseline PHQ-9 (n = 185)	
Baseline PHQ-9 ≥ 10, n (%)	74 (40)^a^
BCT trauma program characteristics	
Received cognitive processing therapy, n (%)	110 (55.28)
Received prolonged exposure, n (%)	89 (44.72)
No. of therapy sessions completed, median [Q1,Q3]	8.00 [6.00,10.00]
Duration of care (week), median [Q1,Q3]	9.57 [7.00,13.57]

*PHQ-9* patient health questionnaire-9, *PCL-5* PTSD checklist for DSM-5, *BCT* blended care therapy, *SD* standard deviation.

^a^Percent among those with PHQ-9 only.

### PTSD symptoms

#### Paired samples t tests

Participants reported significant improvements in PTSD symptoms from pre-to post-treatment. In the entire sample, the mean PCL-5 scores changed from 34.39 to 18.29 (M_D_ = 16.10, SD_D_ = 14.92; *p* < .001; Hedge’s *g* = 1.07; Table 2). Among participants with baseline PCL-5 < 31, mean PCL-5 scores changed from 20.24 to 12.00 (M_D_ = 8.24, SD_D_ = 10.75; *p* < .001; Hedge’s *g* = 0.76). Among participants with baseline PCL-5 ≥ 31, mean PCL-5 scores changed from 45.38 to 23.18 (M_D_ = 22.20, SD_D_ = 14.90; *p* < .001; Hedge’s *g* = 1.48).

**Table 2:** Changes in PTSD and depression symptoms from pre-to-post the BCT trauma program.

Samples	Sample size	Baseline score, mean (SD)	Final score, mean (SD)	Paired differences, mean (SD)	95% CI of the difference	T-value (df)	Hedges’s g
Outcome: PTSD symptoms (PCL-5)							
Entire sample	199	34.39 (15.32)	18.29 (14.76)	16.1 (14.92)	14.01 to 18.18	15.21 (198)***	1.07
Baseline PCL-5 ≥ 31	112	45.38 (10.35)	23.18 (15.4)	22.2 (14.9)	19.41 to 24.99	15.77 (111)***	1.48
Baseline PCL-5 < 31	87	20.24 (6.51)	12.0 (11.14)	8.24 (10.75)	5.95 to 10.53	7.15 (86)***	0.76
Outcome: depression symptoms (PHQ-9)							
Baseline PHQ-9 ≥ 10	74	14.15 (3.69)	8.86 (5.25)	5.28 (5.08)	4.11 to 6.46	8.95 (73)***	1.03

*PHQ-9* patient health questionnaire-9, *PCL-5* PTSD checklist for DSM-5, *CI* confidence interval, *df* degrees of freedom.

***p < 0.001.

#### Reliable improvement, recovery, clinically meaningful improvement

Overall, 165 participants (82.91%) experienced either reliable improvement or recovery in PTSD symptoms, and 129 (64.82%) experienced clinically meaningful improvement (Table 3). Among the 112 participants (56.28%) with PTSD symptoms above the clinical cutoff at baseline, 102 (91.07%) experienced reliable improvement or recovery, and 88 (78.57%) had clinically meaningful improvement in their PTSD symptoms by the end of care.

**Table 3:** Reliable improvement, recovery, and clinically meaningful improvement.

Samples	Sample size	Reliable improvement^a^	Recovery^b^	Reliable improvement or recovery^c^	Reliable improvement and recovery^d^	Clinically meaningful improvement^e^
Outcome: PTSD symptoms (PCL-5)						
Entire Sample	199	164 (82.41)	–	165 (82.91)	–	129 (64.82)
Baseline PCL-5 ≥ 31	112	101 (90.18)	80 (71.43)	102 (91.07)	79 (70.54)	88 (78.57)
Outcome: depression symptoms (PHQ-9)						
Baseline PHQ-9 ≥ 10	74	37 (50.00)	45 (60.81)	51 (68.92)	31 (41.89)	–

*PHQ-9* patient health questionnaire-9, *PCL-5* PTSD checklist for DSM-5.

^a^Reliable improvement: ≥ 5-point decrease on the final PCL-5; or ≥ 6-point decrease on the final PHQ-9.

^b^Recovery: Final PCL-5 < 31 among those with baseline PCL-5 ≥ 31; or final PHQ-9 < 10.

^c^Reliable improvement or recovery: ≥ 5-point decrease on the final PCL-5 or final PCL-5 < 31 among those with baseline PCL-5 ≥ 31; or ≥ 6-point decrease on the final PHQ-9 or final PHQ-9 < 10.

^d^Reliable improvement and recovery: ≥ 5-point decrease on the final PCL-5 and final PCL-5 < 31 among those with baseline PCL-5 ≥ 31; or ≥ 6-point decrease on the final PHQ-9 and final PHQ-9 < 10.

^e^Clinically meaningful improvement: ≥ 10-point decrease on the final PCL-5.

#### Growth curve models

Coefficients for Model 1 are provided in the left column of Table 4. The intercept term (*b* = 25.20, *p* < .001) describes the average PCL-5 score at week 0 for participants reporting subclinical symptoms (i.e., baseline PCL-5 < 31), and the coefficient for the baseline symptom severity (*b* = 21.76, *p* < .001) describes the difference in average PCL-5 scores at week 0 for participants with baseline symptoms in the clinical range (i.e., baseline PCL-5 ≥ 31). The linear effect *Week* (*b* = − 2.53, *p* < .001) indicates that on average, participants exhibited a significant initial decrease in symptoms at a rate of more than 2 units of PCL-5 each week. In addition, the quadratic effect *Week*^2^ (*b* = 0.06, *p* < .001) suggests that the rate of decline diminished over time.

**Table 4:** Growth curve modeling results of PTSD symptoms (PCL-5), b (95% confidence interval).

	Model 1	Model 2
Intercept	25.20 (22.99, 27.41) t = 22.34***	22.29 (19.90, 24.68) t = 18.31***
Baseline PCL-5 ≥ 31 (Ref: PCL < 31)	21.76 (19.09, 24.42) t = 16.00***	26.82 (23.67, 29.96) t = 16.73***
Week	− 2.53 (− 3.09, − 1.98) t = − 8.95***	− 0.81 (− 1.61, − 0.01) t = − 1.99*
Week^2^	0.06 (0.03, 0.10) t = 3.82***	− 0.01 (− 0.06, 0.04) t = − 0.51
Week * Baseline PCL-5 ≥ 31		− 2.99 (− 4.04, − 1.95) t = − 5.64***
Week^2^ * Baseline PCL-5 ≥ 31		0.14 (0.07, 0.20) t = 4.11***
Deviance (− 2*Log likelihood)	10,443.05	10,406.47
Akaike inf. crit	10,465.05	10,432.47
Bayesian inf. crit	10,523.04	10,501.01

PCL-5: PTSD Checklist for DSM-5. Baseline PCL-5 indicator is coded: 0 = PCL-5 < 31, 1 = PCL-5 ≥ 31. Model 1 residual degrees of freedom = 1428, and Model 2 residual degrees of freedom = 1426. Likelihood ratio test: ${\chi }^{2}$(2) = 36.57, *p* < 0.001.

*p < 0.05; **p < 0.01; ***p < 0.001.

**Fig. 1:**
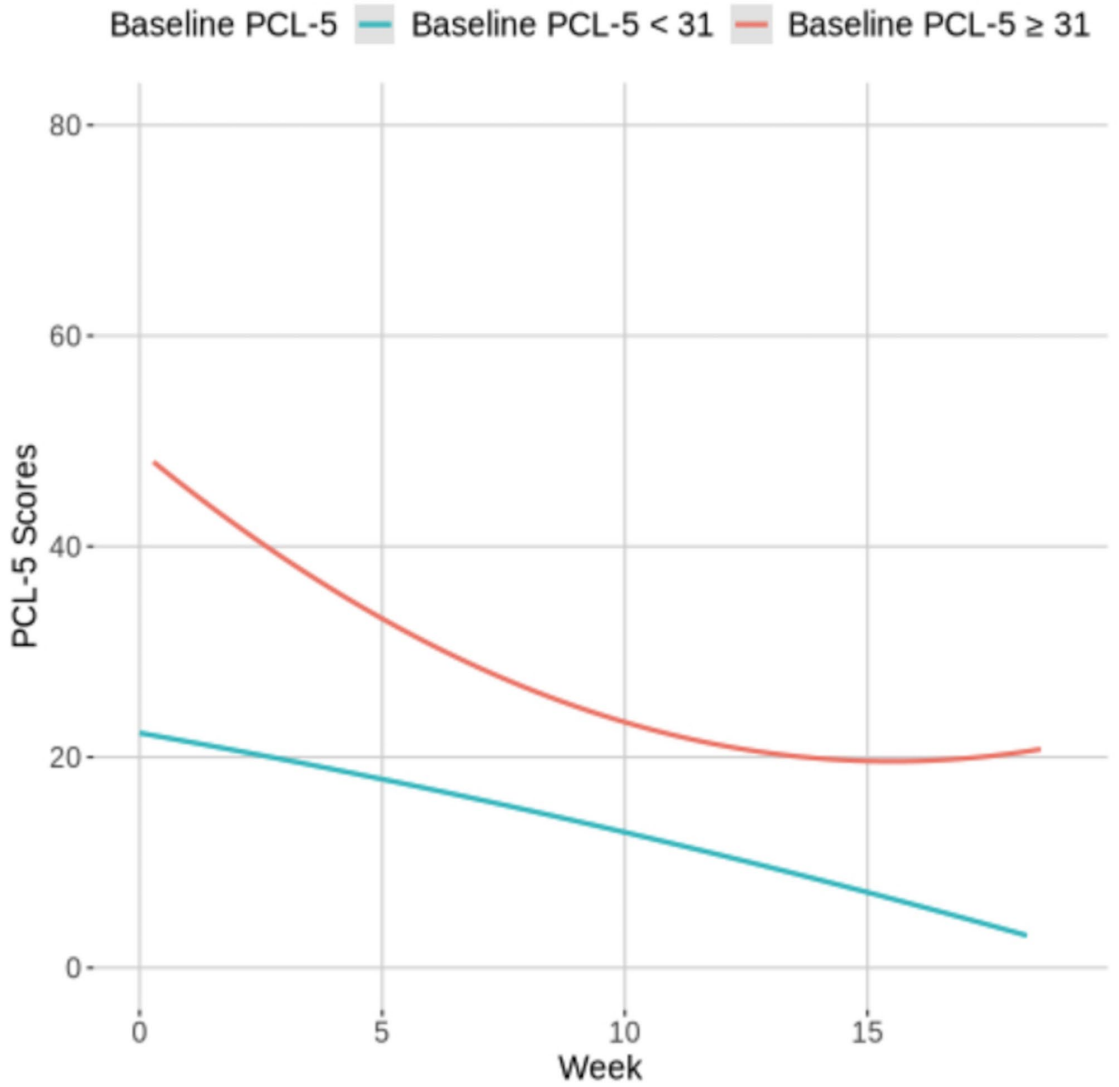
Changes in PCL-5 scores by baseline PCL-5 severity: visual presentation of growth curve modeling results. *PCL-5* PTSD checklist for DSM-5. The growth curve model included baseline PCL-5 severity (PCL-5 ≥ 31 vs. < 31), and its interactions with week and week^2^ as fixed effects.

Coefficients for Model 2 are provided in the right column of Table 4, and Fig. 1 visually presents changes in PCL-5 scores by baseline PCL-5 severity. The presence of a Week*Baseline PCL-5 ≥ 31 interaction (*b*_*Model* 2_ = − 2.99, *p* < .001) indicates that participants with baseline symptoms in the clinical range exhibited a significantly steeper initial decline in PCL-5 scores. In addition, the interaction with the quadratic effect of time was significant (*Week*^2^*Baseline PCL-5 ≥ 31, *b* = 0.14, *p* < .001). The *Week* (*b*_*Model* 2_ = − 0.81, *p* < .05) and *Week*^2^ (*b* = − 0.01, *p* = .611) coefficients describe the predicted trajectory for participants with baseline PCL-5 < 31, demonstrating that PCL-5 scores significantly decreased during each week in care among participants with subclinical symptoms. Follow-up simple slopes analysis^32^ confirmed that the expected change in PTSD symptoms among those with baseline PCL-5 ≥ 31 was characterized by a much steeper initial decline (*Week b* = − 3.81, *p* < .001) and a more rapid recovery trajectory (*Week*^2^ *b* = 0.12, *p* < .001). The inclusion of age, gender, race and ethnicity, and treatment type (CPT or PE) as fixed effects in Model 2 did not improve model fit and were excluded from the final model (Supplementary Table S1). However, Supplementary Fig. S1 provides an exploratory visual presentation of growth curve modeling results of PCL-5 scores over the course of care by treatment type.

### Depression symptoms

Among participants with PHQ-9 data (*n* = 185), 74 (40.0%) reported clinically elevated depression symptoms (PHQ-9 ≥ 10) at the beginning of the BCT Trauma program. The proportion of participants with clinically elevated depression symptoms was significantly higher among participants with baseline PCL-5 ≥ 31 (*n* = 65 out of 105, or 61.90%) compared to participants with baseline PCL-5 < 31 (*n* = 9 out of 80, or 11.25%; McNemar’s *χ*^2^(1) = 9.0, *p* < .001).

In the sub-sample with PHQ-9 ≥ 10 at the beginning of the BCT Trauma program, pre-post improvements were statistically significant (Table 2), with mean PHQ-9 scores changing from 14.15 to 8.86 (M_D_ = 5.28, SD_D_ = 5.08; *p* < .001; Hedge’s *g* = 1.03). In addition, 51 (68.92%) experienced reliable improvement or recovery in depression symptoms (Table 3).

PHQ-9 score changes over the entire course of care were also evaluated by including non-trauma focused therapy sessions before starting the BCT Trauma program. Overall, 100 (50.25%) participants reported elevated depression symptoms at the beginning of treatment (Supplementary Table S2). In this subset, 75 (75.00%) had reliable improvement or recovery by the end of care (inclusive of sessions prior to the BCT Trauma program). The changes in PHQ-9 over the entire treatment were statistically significant, and the mean PHQ-9 scores changed from 15.48 to 7.93 (M_D_ = 7.55, SD_D_ = 5.51; *p* < .001; Hedge’s *g* = 1.36).

## Discussion

This evaluation assessed the clinical outcomes of a BCT program for PTSD symptoms that was offered as a mental health benefit by employers. Overall, 82.91% of participants experienced either reliable improvement or recovery in PTSD symptoms by the end of care, and this proportion was even higher (91.07%) in the subset with clinically elevated PTSD symptoms at the beginning of care. Similarly, participants with clinically elevated PTSD symptoms at baseline exhibited steep initial declines in symptoms during the early stages of treatment that became less steep over time, whereas participants with lower PTSD symptoms at baseline exhibited a more modest rate of decline in symptoms that remained consistent across treatment. These results provide evidence that a BCT Trauma Program can be beneficial among participants presenting with PTSD symptoms, including those whose baseline symptoms are subclinical yet may be experiencing distress related to PTSD symptoms.

Prior research on treatment for PTSD symptoms delivered using a blended care model has reported significant reductions in PTSD and depression symptoms among veterans^30,31^. A primary differentiator of the current study was positioning therapists in the lead of overall care with digital tools and activities supporting client practice, while other studies have primarily focused on digital module-delivered treatment with therapist support. The current study extends the previous literature by evaluating outcomes among participants receiving CPT or PE within a blended care model outside of a VA population. Importantly, in this study, the inclusion of treatment type as a variable in growth curve models did not improve model fit, suggesting either PE or CPT can be effective.

A high rate of participants completed all scheduled therapy sessions in this study (77.39%). In addition, completion rates (i.e., no missed scheduled therapy sessions) were high for both CPT (78.18%) and PE (76.40%). The completion rates observed in this study are in contrast to the literature that has generally reported lower retention for evidence-based PTSD treatments in real-world settings^33,34^. Because greater baseline PTSD symptom severity may contribute to dropout^33,35^, one potential factor explaining the high completion rates in this study could be the inclusion of participants with subclinical levels of PTSD symptoms. At the same time, although research suggests comparable attrition between in-person, face-to-face and video-based delivery of evidence-based PTSD treatment^22^, logistical barriers (e.g., travel) may still contribute to dropout^15,33^. As such, the high completion rates in this study could be at least partially attributable to the remote delivery of this program. It is also a strong possibility that the between-session support provided to clients through digital tools and provider feedback may have encouraged treatment completion. In order to inform real-world efforts to improve completion of evidence-based PTSD treatments, further research is needed to elucidate the factors contributing to the high completion rates observed in this evaluation.

This study suggests video-delivered BCT for PTSD can be an effective approach for delivering evidence-based care for PTSD symptoms. The effect size for pre-post change in PTSD symptoms was large (*g* = 1.07), and even larger in the subset with clinically elevated PTSD symptoms at baseline (*g* = 1.48). The latter effect size, in particular, was comparable to benchmarks estimated from randomized controlled trials of PE and CPT (intent-to-treat *g’s* = 1.38 and 1.69, respectively)^36^. Additionally, in this study the effect size for end-of-care depression outcomes (*g* = 1.03) was comparable to benchmarks for PE and CPT depression outcomes (intent-to-treat *g’*s = 0.97 and 0.91, respectively)^36^. As expected, when assessing depression outcomes over the entire course of care by including standard BCT sessions completed prior to starting the BCT Trauma program, the effect size for pre-post change in PHQ-9 scores over the entire treatment course was even larger (*g* = 1.36).

Strengths of the current study include the evaluation of clinical outcomes under real-world conditions and having an inclusive sample with regards to index trauma as well as to race and ethnicity. This study also assessed PTSD and depression symptoms using validated and reliable measures of symptom severity. Results may also be more generalizable to civilian populations compared with prior evaluations performed only among veterans. However, this study had several limitations. For one, as with any program that serves individuals seeking treatment, regression to the mean effects may also be contributing to some of the observed symptom reduction. In addition, although validated measures were used to assess PTSD and depression symptoms, structured clinical interviews for diagnoses were not conducted. Because the sample size was small and the program was delivered to employed populations and/or their dependents, further research is needed to examine whether results are generalizable. Finally, although consultation groups ensured model fidelity through in-depth session reviews, no formal fidelity measures were used in this evaluation.

In summary, in light of the substantial consequences associated with PTSD, such as the high economic burden (e.g., excess costs of $19,630 per person suffering from PTSD)^6^, increasing the accessibility of evidence-based treatment is essential. This real-world evaluation provides evidence for the clinical benefits of CPT and PE that are delivered completely virtually using a blended care model. These findings provide compelling data to encourage research on the potential role of BCT in improving access to evidence-based care for PTSD symptoms. More specifically, future research could assess whether BCT for PTSD symptoms could be applied to other clinical mental health programs to expand the reach of services to additional populations. Further research is warranted on how to optimize the quantity of care components (e.g., therapy sessions, digital content), particularly across severity levels, and a long-term evaluation to assess the durability of findings. Finally, because it is important to evaluate the scalability of such programs across different contexts, future research should assess outcomes of BCT for PTSD in large-scale, diverse populations.

## Methods

### Design and participants

#### Design

The present study used a retrospective cohort design. Participants were self-referred employees or their dependents who received blended care therapy (BCT) for PTSD symptoms (hereafter referred to as the BCT Trauma program) from Lyra Clinical Associates as an employer-offered mental health benefit. This analysis of de-identified data was determined to be exempt by the WCG Institutional Review Board (Puyallup, WA). All methods were performed in accordance with the relevant guidelines and regulations. Participants provided informed consent to take part in care and have their de-identified data used for research purposes as a part of that consent for care.

#### Participants

**Fig. 2:**
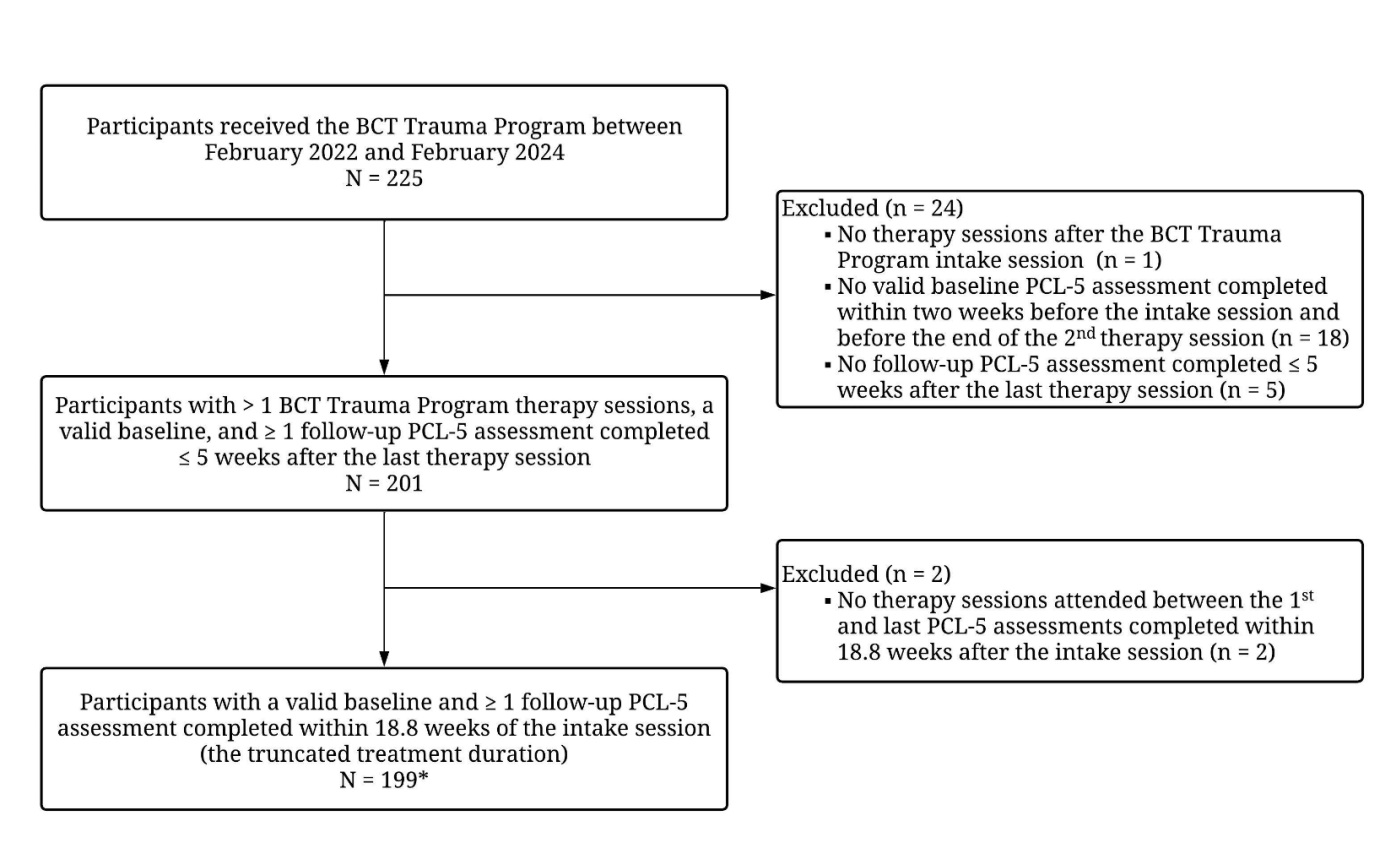
Participant flowchart. *BCT* Blended care therapy. *PCL-5* PTSD checklist for DSM-5. *Of the final sample, 105 (52.76%) received therapy sessions prior to starting the BCT trauma program.

This study included participants who received the BCT Trauma program between February 2022 and February 2024. The initial sample included 225 participants. We excluded 1 participant who did not receive treatment after the intake session, 18 participants who did not complete the first PTSD symptom outcome assessment (i.e., PTSD Checklist for DSM-5 (PCL-5)^37^) within 14 days before the intake session and before the end of the second therapy session, and 5 participants who only submitted one PCL-5 assessment ≤ 5 weeks after the last treatment session (see Fig. 2 for details). To evaluate the impact of the BCT Trauma program over a typical course of treatment, we removed assessments collected > 18.8 weeks after the intake session (corresponding to mean + 1 standard deviation (SD) of the program treatment duration). Participants must also have received ≥ 1 therapy session between the first and last assessments, and as a result, 2 participants were removed. End-of-care assessment scores were the final valid assessment after baseline. The final sample included 199 participants, 105 (52.76%) of whom received non-trauma focused therapy sessions prior to starting the BCT Trauma Program.

### Blended care therapy trauma program

The BCT Trauma Program was provided to clients who had experienced a criterion A traumatic stressor^38^ and were struggling with symptoms of PTSD. During the intake, information regarding the experience(s) of trauma were paired with assessments (e.g., PCL-5, Patient Health Questionnaire-9 (PHQ-9)^39^) to determine severity of PTSD symptoms and evaluate eligibility and fit with the program. The program did not have criteria to exclude participants due to PTSD symptom severity and included those presenting with mild to severe PTSD symptoms. Eligible clients began trauma-specific treatment (i.e., CPT or PE)^40,41^, which comprised live, video-based psychotherapy sessions with a therapist. Based upon the availability of providers in their state of residence, participants were offered either CPT or PE. Treatment was delivered by licensed therapists (e.g., licensed clinical psychologists, licensed clinical social workers, or licensed professional counselors) who were extensively vetted and underwent a BCT Trauma training program.

Modifications to CPT and PE based on existing research suggest the effectiveness of brief protocols^42,43^. Standard length of treatment was set at nine sessions (including the intake), with the flexibility to adjust treatment episode length based on treatment responsiveness in consultation with an expert trauma consultant. Clinicians used CPT, which excludes a trauma narrative. To fit the shortened protocol, the Challenging Beliefs Worksheet (CBW) and an abbreviated list of challenging questions were utilized similar to those supported in research in the CPT treatment manual^40^. Content related to the patterns of problematic thinking were removed, and therapists were encouraged to select three of the five CPT themes (Safety, Trust, Power/Control, Self-Esteem, Intimacy) for the final three sessions. Empirical support for modified CPT protocols can be found in the research literature^42,44^. For PE, modifications included shortening of the duration of the sessions (60 min vs. 90 min), which is supported as non-inferior in the literature^45^, removal of the breathing retraining in the first session, and shortened imaginal exposures (20–25 min vs. 30–45 min) during the actual session time^45^.

Therapy sessions were supported by between-session therapist-client messaging, the assignment of relevant digital tools that clients could access and use between sessions, and therapist-administered feedback on these assignments. Digital tools consisted of digital exercises and assessments that were based on CPT and PE protocols^40,41^. Examples of principles and skills that were reinforced through digital tools include explorations of trauma-related beliefs, understanding traumatic events and traumatic stress, and exposure to trauma-related reminders. Providers could also assign digital video lessons that were developed using transdiagnostic treatment approaches^46–48^, which have been described previously^29^.

Video-based therapy sessions with therapists, digital tools, and assessments were completed on a secure, HIPAA-compliant online platform developed by Lyra Health.

### Blended care trauma training program

A training protocol in line with gold standard models for psychotherapy training programs was implemented as described below^44^. Providers from both groups (PE and CPT) attended a didactic training on trauma assessment (e.g., identifying Criterion A traumatic stressors, using the PTSD Checklist for DSM-5). Providers then attended either the PE or CPT didactic training. Instruction included in-depth explanations of how to implement each psychotherapy with fidelity as well as break-out sessions to practice elements of each therapy. Best practices for implementing PE or CPT using Lyra Health’s blended care digital tools were also discussed.

Providers were then assigned to consultation groups comprised of 1 PE or CPT subject matter expert and 6–7 providers. Consultation groups initially met weekly for 6–8 weeks and then bi-weekly for 2 months. Consultation groups ensured ongoing model fidelity by providing in-depth, ad hoc session reviews.

### Measures

#### PTSD symptoms

The PCL-5 was assigned weekly (before sessions) to assess participants’ self-reported PTSD symptoms^37^. The PCL-5 is a 20-item measure based on the DSM-5 criteria that assesses the presence and severity of PTSD symptoms over the past-month. Responses were rated on a Likert-type scale, ranging from 0 (Not at all) to 4 (Extremely). Items were summed for a total severity score (range: 0 to 80). A PCL-5 score of 31 was previously found to be an optimal cutoff to identify those with a PTSD diagnosis^49^, and the current study used scores ≥ 31 to identify those with clinically elevated PTSD symptoms. Recovery was defined as falling below the clinical threshold at the end of care (final PCL < 31). The minimal threshold for reliable improvement (i.e., reliable change) was 5 points and the threshold for clinically meaningful change was 10 points^50^.

#### Depression symptoms

Based on providers’ assessment of participants’ initial depression symptoms levels, the Patient Health Questionnaire-9 (PHQ-9) was assigned before sessions on a weekly basis (*n* = 185 out of 199 participants)^39^. A score ≥ 10 was considered clinically elevated depression symptoms^39^, and recovery was defined as an end-of-care score below the clinical threshold (final PHQ-9 < 10). A score reduction of ≥ 6 points was considered reliable improvement^51^. End-of-care PHQ-9 scores were the final valid assessments that were completed along with the final valid PCL-5 assessments.

#### Demographics

Participants self-reported age, gender, and race and ethnicity. For analysis, participants who selected more than one category of race or ethnicity were re-categorized as “Multiple,” and those who selected “Native Hawaiian or Other Pacific Islander” or “American Indian or Alaska Native” were re-categorized as “Other” due to small sample sizes.

### Statistical analysis

The clinical impact of the BCT Trauma program on the primary outcomes of PTSD symptoms was evaluated using the following methods: assessing the proportion of participants who exhibited reliable improvement and/or recovery, as well as clinically meaningful improvement; changes in PTSD symptoms from pre- to post-treatment using paired samples t-tests; and changes in PTSD symptoms over the course of treatment using growth curve analysis^52,53^. Participants with at least two valid outcome measures, regardless of program completion status, were included in these analyses.

Specifically, a pair of three-level (assessments nested under participants who were further nested under therapists) growth curve models were examined. Model 1 included linear (*Week*) and quadratic (*Week*^2^) fixed effects representing the amount of time between the intake session (*Week* = 0) and each PCL-5 assessment, as well as a random intercept and random effects of the linear and quadratic terms of time for participants. Because pre-treatment PTSD symptom severity may be linked to the strength of treatment outcomes^54^, baseline PTSD symptom severity represented by a dummy-coded indicator (i.e., 0 = baseline PCL-5 < 31; 1 = baseline PCL-5 ≥ 31) was also included as a fixed effect. Model 2 incorporated the interaction terms involving participants’ baseline severity and the two effects of time (linear, quadratic). An additional model was examined by adding participants’ age, gender, race and ethnicity, as well as the treatment type received (CPT or PE) as fixed effects to Model 2.

As a secondary outcome, among participants with clinically elevated depression symptoms at baseline (PHQ-9 ≥ 10), the proportion who achieved reliable improvement and/or recovery on the PHQ-9 was calculated and mean changes from pre-to post-treatment were evaluated using paired samples t-tests. As previously mentioned, prior to starting the BCT Trauma program, more than half of participants (*n* = 105; 52.76%) received non-trauma focused therapy sessions. Therefore, we also evaluated changes on the PHQ-9 across the entire treatment period, inclusive of prior BCT received (See Supplementary Table S2). Only baseline PHQ-9 scores submitted within 14 days of the first therapy session and before the second therapy session were included in the analysis of changes in PHQ-9 over the entire treatment period.

All analyses were conducted using R *4.2.3*, and growth curve models were fitted using version 1.1.34 of the lmer library using full maximum likelihood estimation^55^.

## Electronic Supplementary Material

Below is the link to the electronic supplementary material.

Supplementary Material 1

## Data Availability

All data supporting the findings are presented in the article and supplementary tables. As a business associate under HIPAA, Lyra Health is only permitted to use patient information as outlined in our Business Associate Agreements with our customers, which are covered entities. Many of Lyra Health’s customer agreements do not permit disclosure of de-identified patient data. Therefore, because of contractual restrictions, de-identified datasets cannot be shared.
