# Supplementary material for: Real-world evaluation of an evidence-based telemental health program for PTSD symptoms
Source: Sci Rep. 2025 Jan 3;15:730. doi: 10.1038/s41598-024-83144-6 (PMC11698986; doi:10.1038/s41598-024-83144-6)
Supplement: Supplementary file 1 — Supplementary Material 1 [file 41598_2024_83144_MOESM1_ESM.docx]

**Supplementary Table S1.** Growth Curve Modeling Results of PTSD Symptoms (PCL-5), b (95% Confidence Interval)

|  | Model 2 | Model 2 + Demographics & Treatment Type |
| --- | --- | --- |
| Intercept | 22.29 (19.90, 24.68)  t = 18.31*** | 24.71 (19.18, 30.25)  t = 8.75*** |
| Baseline PCL-5 ≥31 (Ref: PCL-5<31) | 26.82 (23.67, 29.96)  t = 16.73*** | 26.83 (23.73, 29.93)  t = 16.96*** |
| Week | -0.81 (-1.61, -0.01)  t = -1.99* | -0.78 (-1.59, 0.02)  t = -1.91 |
| Week² | -0.01 (-0.06, 0.04)  t = -0.51 | -0.02 (-0.07, 0.03)  t = -0.66 |
| Week * Baseline PCL-5≥31 | -2.99 (-4.04, -1.95)  t = -5.64*** | -2.99 (-4.04, -1.95)  t = -5.62*** |
| Week² * Baseline PCL-5≥31 | 0.14 (0.07, 0.20)  t = 4.11*** | 0.14 (0.07, 0.20)  t = 4.11*** |
| Age |  | -0.02 (-0.16, 0.12)  t = -0.24 |
| Gender (Ref: Female) |  |  |
| Male |  | -2.71 (-6.71, 1.30)  t = -1.32 |
| Other/Missing |  | -1.51 (-8.80, 5.79)  t = -0.41 |
| Race & Ethnicity (Ref: White) |  |  |
| Hispanic or Latino |  | 0.59 (-3.68, 4.86)  t = 0.27 |
| Asian or Pacific Islander |  | -7.03 (-12.25, -1.81)  t = -2.64** |
| Black or African American |  | 0.54 (-5.77, 6.85)  t = 0.17 |
| Multiple |  | -1.99 (-5.88, 1.91)  t = -1.00 |
| Other |  | -2.69 (-8.79, 3.41)  t = -0.86 |
| Prefer not to disclose/Unknown |  | 2.16 (-6.50, 10.81)  t = 0.49 |
| Treatment Type (Ref: Cognitive Processing Therapy) |  |  |
| Prolonged Exposure |  | -1.63 (-4.31, 1.06)  t = -1.19 |
| **Deviance (-2*Log likelihood)** | 10406.47 | 10392.94 |
| **Akaike inf. crit.** | 10432.47 | 10438.94 |
| **Bayesian inf. crit.** | 10501.01 | 10560.19 |

**Notes.** PCL-5: PTSD Checklist for DSM-5. Baseline PCL-5 indicator is coded: 0 = PCL-5 < 31, 1 = PCL-5 ≥ 31. Model 2 residual degrees of freedom = 1426.  Model 2 + demographics & treatment type residual degrees of freedom = 1416. Likelihood ratio test: χ2(10) = 13.53, *p* = 0.196.

* *p* < 0.05; ** *p* < 0.01; *** *p* < 0.001

**Supplementary Table S2**. Inclusion of Depression Outcomes at the beginning of any BCT: Baseline Characteristics and Depression Symptom Outcomes

| **Participant Characteristics** | |
| --- | --- |
| Received ≥1 Therapy Session Before Starting the BCT Trauma Program, n (%) | 105 (52.76) |
| No. of therapy sessions completed before starting the BCT Trauma program, median [Q1,Q3] | 2.00 [1.00,3.00]ᵃ |
| **Outcome: Depression Symptoms (PHQ-9)ᵇ^,^ᶜ (N = 100)** | |
| Reliable improvement or Recovery ᵈ, n (%) | 75 (75.00) |
| Baseline score, Mean (SD) | 15.48 (3.76) |
| Final score, Mean (SD) | 7.93 (5.19) |
| Paired differences, Mean (SD) | 7.55 (5.51) |
| 95% CI of the differences | 6.46 to 8.64 |
| T-value (df) | 13.7 (99) *** |
| Hedge's g | 1.36 |

**Notes**: BCT: Blended care therapy; PHQ-9: Patient Health Questionnaire-9.

^a^Calculated among participants with ≥1 therapy session before starting the BCT Trauma program

^b^Calculated among participants with PHQ-9 ≥ 10 at the beginning of any BCT (i.e., standard BCT and/or BCT for Trauma).

^c^Baseline PHQ-9 was defined as the earliest available valid PHQ-9 score; final PHQ-9 were those collected at the end of the BCT Trauma program

^d^Reliable Improvement:  ≥ 6-point decrease on the final PHQ-9 among those with baseline PHQ-9 ≥ 10; Recovery: Final PHQ-9 < 10 among those with baseline PHQ-9 ≥ 10

*** *p* < 0.001

**Supplementary Figure S1**. Changes in PCL-5 Scores by Treatment Type (CPT or PE): Visual Presentation of Growth Curve Modeling Results


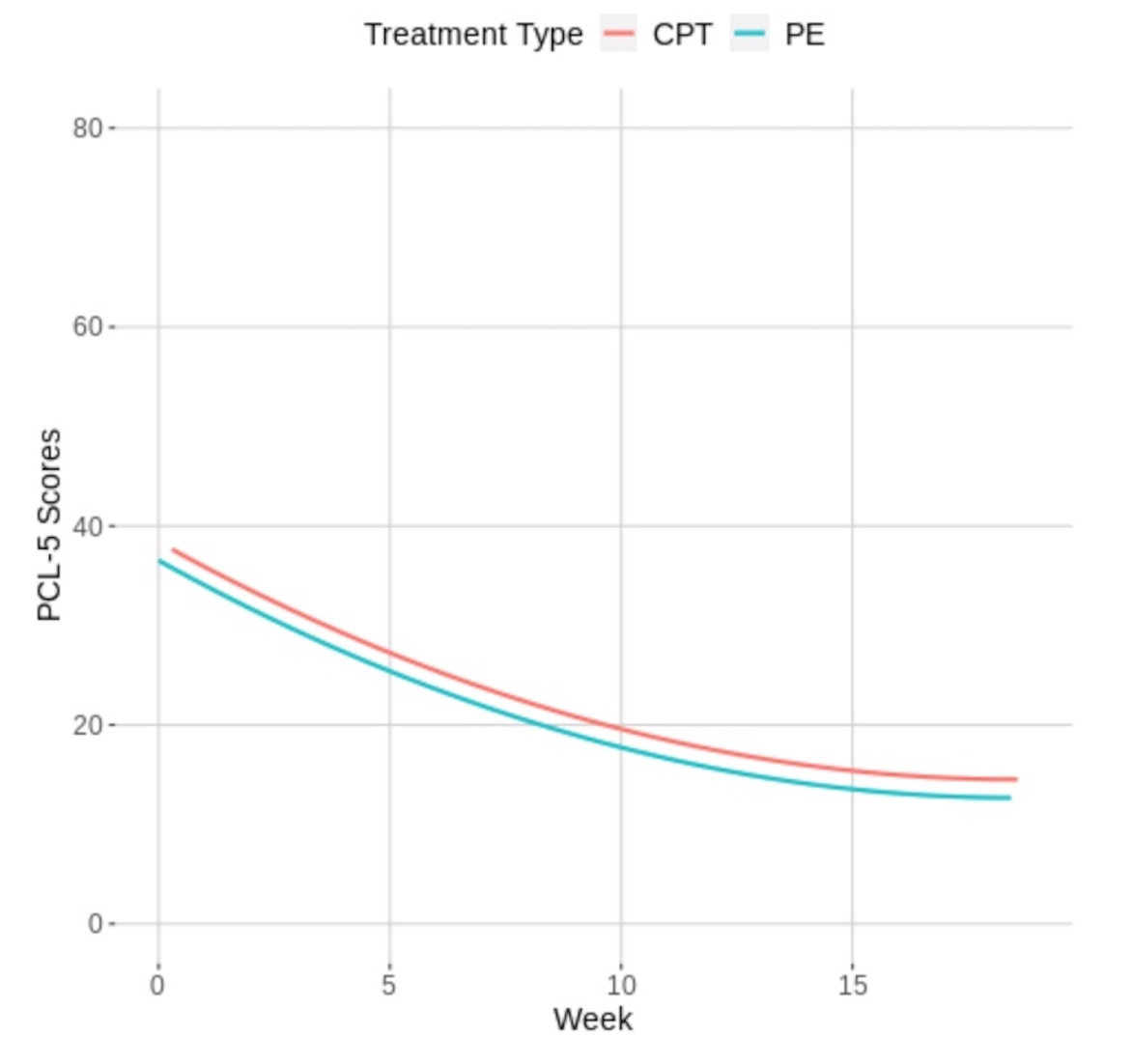


**Notes**. CPT: Cognitive Processing Therapy; PE: Prolonged Exposure; PCL-5: PTSD Checklist for DSM-5. The growth curve model included week, week^2^, and treatment type (CPT versus PE) as fixed effects.
